# Treatment of life-threatening wounds with a combination of allogenic platelet-rich plasma, fibrin glue and collagen matrix, and a literature review

**DOI:** 10.3892/etm.2014.1747

**Published:** 2014-05-29

**Authors:** MEHDI ASADI, DARYOUSH HAMIDI ALAMDARI, HAMID REZA RAHIMI, MOHSEN ALIAKBARIAN, ALI JANGJOO, ABBAS ABDOLLAHI, MOSTAFA MEHRABI BAHAR, ALI AZADMAND, NASER FORGHANI, MOHAMMAD NORI SADEGH, MOHAMMAD ESMAIL KHAYAMY, ALEXANDER SEIFALIAN

**Affiliations:** 1 Surgical Oncology Research Center, Imam Reza Hospital, Faculty of Medicine, Mashhad University of Medical Sciences, Mashhad 917794-8564, Iran; 2 Stem Cell and Regenerative Medicine Research Group, Department of Biochemistry, Stem Cell Laboratory, Faculty of Medicine, Mashhad University of Medical Sciences, Mashhad 917794-8564, Iran; 3 Student Research Committee, Department of Modern Sciences and Technologies, Faculty of Medicine, Mashhad University of Medical Sciences, Mashhad 917794-8564, Iran; 4 Blood Transfusion Research Center, High Institute for Research and Education in Transfusion Medicine, Mashhad 91875, Iran; 5 UCL Centre for Nanotechnology and Regenerative Medicine, Division of Surgery and Interventional Science, University College London, Royal Free Hampstead NHS Trust Hospital, London, UK

**Keywords:** recalcitrant wounds, platelet-rich plasma, fibrin glue, collagen matrix

## Abstract

Currently there is no ideal procedure for the treatment of recalcitrant ulcers that are unresponsive to the majority of common treatments. However, several novel approaches have been proposed, including bone marrow stem cells, platelets, fibrin glue and collagen matrix. For the first approach treatment of a chronic wound, a non-invasive method is highly desirable. The present study was undertaken with the aim of evaluating the effect of a combination of platelets, fibrin glue and collagen matrix (PFC) in one treatment. A total of ten patients with aggressive, refractory, life-threatening wounds were recruited for the study and their treatment effects were evaluated. Initially, the ulcers were extensively debrided, measured and photographed at weekly intervals. The PFC combination was applied topically to the wound every two days. Following treatment, the wound was completely closed in nine patients and was markedly reduced in the other patient. The mean 100% healing time for the nine patients was 11.3±5.22 weeks. There was no evidence of local or systemic complications or any abnormal tissue formation, keloid or hypertrophic scarring. Therefore, the results of the present study indicate that in the first approach, the combination of PFC components may be used safely in order to synergize the effect of chronic wound healing.

## Introduction

Recalcitrant wounds are associated with increased morbidity and mortality, have a negative impact on the patient quality of life and pose a serious burden on the health care system (1,2). The cost of treating non-healing wounds has been estimated to be $70,000 (3). Considering other indirect costs, including absence from work, loss of employment, cost of medical transport, assistance with daily living and self-care and medication expenditures, it has been estimated that almost $5 billion is spent annually in the USA on ulcer treatment (4). Therefore, promoting the acceleration of wound healing is highly desirable since patient quality of life is likely to improve and the economic impact on the health care system is likely to reduce.

The management of recalcitrant ulcers is a major challenge clinically. Current therapies include debridement, offloading and supplementary treatments. However, the response to treatment is often poor and the outcome disappointing. These wounds place a limb at risk of infection and amputation and also puts patients at risk of mortality. Considering that optimum wound healing requires the well orchestrated integration of complex biological and molecular events involved in cell migration, proliferation, extracellular matrix deposition and remodeling (5), several novel approaches have been proposed for recalcitrant ulceration treatment. These include the use of stem cells (6), platelet-derived growth factors (7) and fibrin glue (8). Each of these approaches has been reported to increase the response time of healing chronic wounds.

In the present study, a combination of allogeneic platelets, fibrin glue and collagen matrix (PFC) was used for the treatment of life-threatening wounds.

In platelets, the bioactive factors are located in the α-granules and dense granules. In tissue regeneration, the wound heals through three phases: Inflammation, proliferation and remodeling. The bioactive factors are active during each of these phases. The α-granules contain growth factors and cytokines, including transforming growth factor-β, platelet-derived growth factor, insulin-like growth factor I and II, fibroblast growth factor, epidermal growth factor, vascular endothelial growth factor and endothelial cell growth factor. These growth factors and cytokines are important in cell proliferation, chemotaxis, cell differentiation and angiogenesis. The dense granules contain serotonin, histamine, dopamine, calcium and adenosine. These non-growth factors have fundamental effects on the biological aspects of tissue repair. Histamine and serotonin increase capillary permeability, which allows inflammatory cells greater access to the wound site and activates macrophages. Polymorphonuclear leukocytes migrate towards the area of inflammation, and soon thereafter, cells begin to proliferate while fibroblasts aid the formation of a ground substance. Adenosine receptor activation modulates inflammation during wound healing (10). Thus, the bioactive factors play a central role in the healing processes by modulating the recruitment, duplication, activation and differentiation of various cell types. Platelets are used in the form of platelet rich plasma (PRP) which is prepared in a two-step process. Whole blood is initially centrifuged to separate the plasma from the red blood cells and centrifuged again in order to separate the PRP from the platelet-poor plasma (11). Clinically valuable PRP contains at least one million platelets per μl (4–5 times more than the blood base line) (12). Lesser concentrations may not be relied on to enhance wound healing, and greater concentrations have not been shown to increase wound healing (13).

Fibrin glue is a topical biological adhesive, the effect of which mimics the final stages of coagulation, wherein thrombin splits off fibrinopeptide A and B from the fibrinogen chain to form a monomer, which polymerizes to form a fibrin clot at the site of application. Fibrin glue is a promising adjunct treatment in numerous surgical fields and is beneficial in procedures which involve a high risk of postoperative bleeding or the leakage of air, blood and other fluids (14). Fibrin clots provide an important temporary extracellular matrix for wound healing. Therefore, fibrin glue was used to apply an admixture of platelets to the wound. The structural composition of fibrin and the binding of fibrin to cells and proteins determines the wound healing process. This represents an ideal delivery vehicle for additional cells for the treatment of chronic wounds (8).

Collagen matrix functions as a scaffold for regeneration. When applied to a tissue defect, the sprouting of capillaries and the migration of fibroblasts into the collagen results in the induction of angiogenesis and fibroplasia. The efficacy of the collagen matrix has been demonstrated for the treatment of deep sacral ulcers (9).

In the present study, ten patients were recruited. The aim of the study was to evaluate the treatment of recalcitrant wounds using a combined application of PFC as a delivery vehicle for the sustained release of platelet-fibrinogen rich plasma (PFRP)-derived bioactive factors to stimulate healing in recalcitrant ulcers, where conventional treatment methods have failed. To the best of our knowledge, this is the first time that this combination has been applied for the treatment of chronic wounds.

## Patients and methods

### Patients

In total, ten patients with life-threatening recalcitrant ulcers, that did not respond to any conventional therapy, were included in this study. The patients exhibited various wound categories, including deep (undermined wounds, 4 cases; tunneling wounds, 5 cases) and superficial wounds (1 case). The study was conducted in accordance with the principles of the 1996 Declaration of Helsinki, with good standards of clinical practice. The study protocol, informed-consent forms and other study-related documents were reviewed and approved by the Human Research Ethics Committee of Mashhad University of Medical Sciences (Mashhad, Iran). All patients were able to read and understand, and willingly signed the informed-consent form for their participation in the study. Inclusion criteria included the presence of a recalcitrant wound (diabetic, vascular, compression or traumatic) for a minimum of three months. In addition, the patients had to be >18 years of age (both genders) and had to agree to comply with the protocol requirements, including the self-care of wounds and all follow-up visit requirements. Patients were excluded if they were pregnant, lactating mothers or were receiving or had received chemotherapy (within eight weeks of the study screening visit). In addition, patients were excluded if they were current participants in an additional clinical investigation or current candidates for vascular surgery, angioplasty or stenting. Patients presenting with the clinical characteristics of cellulitis at the ulcer site, purulence or sinus tracts that were unable to be removed by debridement of the wound, malignant wounds, vasculitis or connective tissue disease, bone marrow involvement (lymphoma-leukemia) and any systemic infection, or those being treated with corticosteroids were also excluded.

### Platelet preparation

Platelets were prepared at the Blood Transfusion Organization (Mashhad, Iran). Great care was taken to exclude any contamination with the red blood cells, thus, only pure platelets that were well within their active period and were qualified, according to viral safety tests in accordance with blood transfusion regulations, were used.

The platelets and fibrin glue were prepared according to standard procedures. Following the collection of 400 ml peripheral blood from the ABO match donor and transferring the sample to commercial 450-ml triple blood donation bags, the platelets were initially prepared by centrifugation at 2,000 × g for 2 min and a second centrifugation at 4,000 × g for 8 min. Subsequently, the supernatant plasma was separated and 25 ml PRP remained (15). The fibrinogen concentrate was prepared from separated plasma by two biochemical methods (16), the cryoprecipitation or the ethanol precipitation method. For the cryoprecipitation method, following a −70°C freeze and a 4°C thaw, the plasma was centrifuged at 6,500 × g for 5 min. The supernatant plasma was removed to leave a final volume of 25 ml. In the ethanol precipitation method, absolute ethanol at 0°C was added to the plasma (10% v/v) and fibrinogen was obtained by centrifugation at 6,500 × g for 15 min. The supernatant plasma was removed to leave a final volume of 25 ml. Concentrated fibrinogen (25 ml) was mixed with the platelets to form PFRP at a final volume of 50 ml. Thrombin (1 ml) was prepared from the removed plasma by adding 10% calcium gluconate. Viral inactivation was performed for PFRP and thrombin by heating at 62°C for 1 h. The samples were then frozen at −20°C until required (for a maximum of three months). Prior to the application of PFRP, the necrotic and devitalized wound area was surgically debrided until the bleeding was recognized macroscopically. This allowed the PFRP to come into contact with viable wound tissue. When used, 50 ml PFRP was mixed with 1 ml thrombin and calcium gluconate. The collagen matrix (Surgicoll^®^; MBP, Medical Biomaterial Products, GmbH, Neustadt-Glewe, Germany) was immediately impregnated with this 51-ml PFRP solution and placed on the wound. Finally, paraffin gauze pads were placed over the wound and a bolster of rolled gauze pads were placed over the paraffin gauze. The dressing was subsequently wrapped with a rolled gauze. After two days, the entire dressing was removed and the wound was irrigated with isotonic sodium chloride solution. The wound was treated every two days, as aforementioned, for the formation of granulation tissue and closure. The patients were followed-up regularly for ulcer closure and any other possible complications. Images were captured on a digital camera on day 0 and at every three weeks, until the wound had healed. In certain cases, the ulcer volume was measured on day 0, according to the volume of normal saline which filled the ulcer. In other cases, the ulcer dimensions (length × width × depth) were measured on day 0. The effectiveness of PFRP-collagen application was evaluated after two months and in case of healing, the treatment continued until the wound closed.

## Results

### Overall analysis

**Figure 1:**
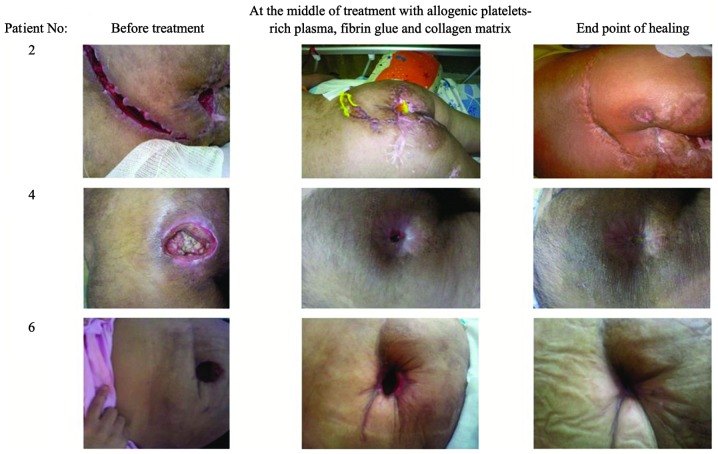
Images of wounds from certain patients prior to and following treatment with allogenic platelet-rich plasma, fibrin glue and collagen matrix.

Patient characteristics, medical history, wound size and the duration of the wound are presented in Table I. Following treatment, the wound was completely closed in nine patients and markedly reduced in the other patient. The mean 100% healing time for nine patients was 11.3±5.22 weeks and the mean patient age was 45.1±1.03 years. Overall, 70% of patients were male. There was no evidence of local or systemic complications or any abnormal tissue formation, keloid or hypertrophic scarring. Images of the wounds, prior to and following treatment, are presented in Fig. 1.

## Discussion

In the present study, the effects of PFC combination treatment on ten patients with recalcitrant and life-threatening wounds were evaluated. The patients were critically ill with high fevers (even with the administration of antibiotics) and had not responded to traditional treatment modalities, including debridement, offloading and complementary therapies, such as antibiotic therapy, blood glucose level control, administration of zinc sulfate and multivitamins and the irrigation of the wound with normal saline and dressings. However, following treatment with the combination described, nine of the ten wounds were completely healed while the rest had improved markedly. In all the wounds, the ulcer bed rapidly demonstrated marginal in-growth, granulation tissue development, neovascularization and epithelialization. The appearance of ulcer bed bleeding during dressing changes and ulcer palpation was observed and was deemed to be a positive indicator for wound healing.

In the present study, a control group was not selected since there was not a logical comparison to the patient group. The patients did not respond to any conventional or traditional therapies and the severe wound put the patient’s life at risk, each patient was the self-control for themselves.

Although autologous and allogeneic PRP therapy is currently used clinically to stimulate tissue growth and regeneration and has been demonstrated to be effective in accelerating repair in chronic skin wounds (17,18), the consensus on the therapeutic use of PRP remains controversial. In the literature, the majority of studies are performed on patients whose wounds have failed to heal with the use of conventional treatment techniques.

In the first clinical study, Knighton *et al* (1986) demonstrated that the topical application of growth factors promoted the healing of chronic cutaneous ulcers (7). Autologous platelet-derived wound healing factors (PDWHF) were used to treat 49 patients with chronic non-healing cutaneous ulcers. A multivariant analysis revealed a direct correlation between 100% healing with initial wound size and the initiation of PDWHF therapy (7).

In a prospectively randomized, blind trial by Knighton *et al* (19), 32 patients with chronic, non-healing, cutaneous wounds of the lower extremities were randomized and treated for eight weeks with autologous PDWHF or placebos, the end point of the study was the epithelialization of the wound. The results revealed that in the group who received treatment, 81% of patients exhibited epithelialization in eight weeks compared with 15% in the control group (P<0.0001) (19). In addition, the results demonstrated a highly statistically significant effect of topically applied PDWHF on the repair of chronic, non-healing, cutaneous ulcers (19).

In a pilot study by Atri *et al* (20), homologous platelet-derived wound healing factors (HPDWHFs) were used to treat recalcitrant ulcers in 23 patients with 27 persistent non-healing ulcers. The patients were initially subjected to controlled wound care for three months, with saline solution and silver sulfadiazine dressings. At the end of this period, persistent non-healing ulcers were treated by topical administration of HPDWHFs and silver sulfadiazine. Ulcer parameters were recorded on the first day and every week throughout the therapy until complete epithelization was achieved in either group. Each ulcer acted as its own control. In the controlled wound care group, only three ulcers in three patients achieved complete healing; the remaining 24 ulcers in 20 patients failed to achieve even 50% healing in the stipulated three-month period. However, when subjected to HPDWHF applications, these ulcers healed completely, with 100% healing occurring in 9.67±4.9 weeks (range, 3–19 weeks), which was statistically significant (P<0.01). The healing response to HPDWHF applications was of uniform progression over the weeks. Only the basic cause of the ulcer determined the healing rates in this group. The shortest and the longest time to achieve 100% healing occurred in patients with diabetes (6.88±2.97 weeks) and in the venous stasis group (14.00±7.07 weeks) (20).

In a retrospective cohort study (with certain limitations) by Margolis *et al* (21), the effectiveness of autologous platelet releasate was assessed for the treatment of diabetic neuropathic foot ulcers in 26,599 patients. The results demonstrated that platelet releasate was more likely to be used in more severe wounds and was more effective in treating these wounds than the standard treatment.

In a prospective non-blinded study with 24 patients, Crovetti *et al* (2004) demonstrated the efficacy of once-weekly applications of autologous (three patients) or homologous origin (21 patients) platelet gel (PG) in healing cutaneous chronic wounds with various etiologies, including diabetes-related, vascular insufficiency, infectious disease, post-traumatic, neuropathic and vasculitis-related. At the time of publication, nine patients had healed completely, two patients had received cutaneous grafts, four patients had stopped treatment and nine patients had responded partially and were continuing to receive treatment (22). Although pain was reportedly reduced with the application of PG, neither patients nor clinicians were blinded to the treatment, possibly introducing bias to the self-report of pain (22).

A prospective, randomized, controlled multicenter trial in the USA by Driver *et al* (23), involving 40 patients with type 1 and type 2 diabetes (19 patients in the PRP group and 21 patients in the control group), reported the use of autologous PRP for the treatment of diabetic foot ulcers. The authors identified that 68.4% of patients in the PRP group (mean healing time, 42.9±18.3 days) and 42.9% of patients in the control group (mean healing time, 47.4±22.0 days) had wounds that healed (23).

A case study by McAleer *et al* (24), regarding a 57-year-old male with type 2 diabetes and a wound of six months duration, reported that the weekly use of autologous PRP was successful in healing a chronic lower extremity wound after four weeks.

In the case study by Knox *et al* (25), a 55-year-old male with a chronic non-healing decubitus ulcer of the sacrum, >1 year in duration, was treated with autologous PRP. The introduction of PRP therapy at week 14 led to a 26% reduction in wound depth over four weeks. At week 19, PRP therapy was combined with a powdered skin substitute to create a platelet-rich tissue graft. The combination led to marked results, eliminating wound tunneling and reducing the wound dimensions from 6.2×6.7×2.7–5.0×6.0×1.4 cm (length × width × depth) (25).

In the case report by Ficarelli *et al* (26), a chronic venous leg ulcer (8×5 cm with raised margins) of a 79-year-old female almost completely healed following 20 allogenic PRP applications. Complete healing was observed one month following discontinuation with treatment.

A a pilot study by O’Connell *et al* (27), comprised a total of 21 patients, 12 patients with 17 venous lower-extremity ulcers and nine patients with 13 non-venous lower-extremity ulcers. The chronic lower-extremity ulcers were treated with autologous platelet-rich fibrin matrix membrane (PRFM). The primary endpoints were the incidence and time to complete closure, while the secondary endpoints were the incidence and time to 75% closure. Complete healing was achieved in 66.7% of the patients with venous lower-extremity ulcers in 7.1 weeks (median, six weeks) following an average of two applications of PRFM per patient. Of the non-venous lower extremity ulcer group, 44% of patients treated with PRFM healed completely during the study period (27).

In a perspective trial by Jeong *et al* (28) using 100 patients with diabetic foot ulcers, 52 patients were treated using a blood bank platelet concentrate and 48 patients (control group) were treated with topical fibrinogen and thrombin. Complete wound healing was achieved in 79% of the blood bank platelet concentrate-treated group and 46% of the control group (P<0.05). The times required for complete healing were 7.0±1.9 and 9.2±2.2 weeks in the blood bank platelet concentrate-treated and control groups, respectively (P<0.05). The degrees of wound shrinkage were 96.3±7.8 and 81.6±19.7% for the treated and control groups, respectively (P<0.05). No adverse events associated with the study treatment were observed (28).

In a pilot study by Chen *et al* (29) involving 15 patients with 17 recalcitrant ulcers of various etiologies, the safety and efficacy of a combining allogeneic single-donor platelet and fibrin glue to enhance skin graft take for treating ulcers was evaluated. It was observed that the majority of the skin grafts were successful. The interval between the skin graft and complete wound healing ranged between 3 weeks and 2 months. No adverse reactions or recurrence of ulcers were observed during the three- to 18-month follow-up period (29).

In a pilot study by Marinacci *et al*, 7 patients were diagnosed with a diabetic ulcer with an extension of >3.5 cm^2^. The ulcers were treated using autologous platelet and fibrin gel. Overall, four patients achieved total recovery of the ulcers, while three patients experienced a >60% reduction in the ulcer diameter. The authors hypothesized that the recovery of the ulcers was associated with platelet activation in the specific ulcer area (21).

Sell *et al* (30) treated chronic stage IV pressure ulcers, in three veterans with spinal cord injury (SCI), with a sustained release PRP therapy to stimulate wound healing. PRP treatment consistently resulted in the formation of granulation tissue and improved the vascularity for each of the three patients, while reducing the overall ulcer area and volume. It was observed that the controlled release of growth factors from PRP has a positive stimulatory effect on the healing rate of chronic pressure ulcers in individuals with SCI (30).

A study by Greppi *et al* (31) on 11 hypomobile elderly patients with 14 chronic skin ulcers, who were unable to undergo autologous blood processing and had previously been ineffectively treated with expensive advanced medications for 8–275 weeks, evaluated the clinical efficacy of allogeneic PG prepared with standard blood banking procedures from routine platelet concentrates (PCs) obtained from buffy coats. No improvement was observed in three patients with 24, 27 and 30 cm^3^ ulcers who were unable to be treated for any longer than 4, 7 and 8 weeks due to progressively worsening clinical conditions. By contrast, 11 ulcers with a median size of 3.2 cm^3^ (range, 0.2–3.6 cm^3^) in the remaining 8 patients demonstrated 91±14% reduction following a median of 12 weeks (range, 1–20 weeks). The cost of PG treatment (€19,976) amounted to ~10% of the ineffective advanced medication hospital reimbursement fees (€191,236). This study demonstrated the efficacy and feasibility of allogeneic PG for the treatment of recalcitrant ulcers in elderly hypomobile patients for whom autologous blood processing may be difficult (31).

Contrary to the aforementioned studies, in a randomized, prospective, double-blind, placebo-controlled study of topical autologous PDWHF in 18 patients [eight patients with nine wounds were treated with a placebo solution (controls) and 10 patients with 17 wounds were treated with PDWHF (treatment group)], Krupski *et al* (32) failed to demonstrate that autologous PDWHF provided additional benefit over traditional therapy for healing chronic non-healing cutaneous wounds.

It has been reported that collagen significantly promotes the repair process, particularly in the early stages. Collagen is used clinically with bone marrow stem cells by plastic surgeons in Japan for the treatment of chronic and acute wounds as a scaffold biomaterial (33–35).

In the pathophysiology of chronic non-healing wounds, the following causative factors have been documented: i) Significantly decreased local concentration, stability and bioavailability of bioactive factors; ii) significantly higher local activity of matrix metalloproteinases that degrade the extracellular matrix, impair tissue repair and suppress cell proliferation and angiogenesis (35,36); and iii) phenotypically altered and/or senescent mesenchymal cells that fill the dermis of the skin (37). Accordingly, correction of the concentration of bioactive factors and wound matrix and reconditioning of phenotypically altered resident cells may lead to the success and efficacy of chronic wound healing.

In this study, rational beyond using PRP that it replenish the significantly decreased local concentration, stability and bioavailability of bioactive factors. PRP play a great role in host defense mechanism at the wound site by producing signaling proteins that attract macrophages (38), it also has anti-microbial activity against *Escherichia coli* and *Staphylococcus aureus*, including methicillin-resistant *Staphylococcus aureus* (39), *Candida albicans* (40) and *Cryptococcus neoformans* (40). Fibrin glue, beyond behaving as a provisional matrix, actively recruits cells to trigger fibrin-mediated responses, including cell adhesion, migration, proliferation and tubule formation. Fibrin also promotes cell growth and vessel formation, which are beneficial during wound repair. Collagen has two roles, firstly functioning as a scaffold and secondly functioning as a sustained release method for the delivery of PRP growth factors and cytokines which are highly advantageous in the chronic wound state where senescent cells are prevalent (4) and may benefit from the sustained presence of stimulating factors.

In the present study, the patients required long-term treatment with platelets, for which large volumes of blood must be aspirated from patients. However, this was not feasible, thus, the allogenic platelets were replaced. All safety tests were performed for blood bank products, whilst paying attention to potential safety concerns. In addition to negative viral tests for the platelets which were used on the patients, viral deactivation was performed by heating.

According to the study by Ravari *et al* (41), it was hypothesized that for those patients who did not respond to the PFRP therapy, considering the pathological factors associated with the phenotypically altered and/or senescent mesenchymal cells in the wound, stem cell therapy should be considered as a second approach for the treatment of recalcitrant wounds. This hypothesis requires further study.

In conclusion, the results of the present study demonstrate the feasibility of the proposed practical model as a non-invasive method in the first approach for the treatment of life-threatening wounds. The use of PFRP-collagen therapy, involving a combination of sustained and immediate release of growth factors, healed or significantly reduced the wound size in the various categories of recalcitrant ulcer, including deep (undermined and tunneling wounds) and superficial wounds. There was no evidence of local or systemic complications associated with the procedure. Thus, on the basis of these results, the authors are currently performing a larger study with the aim of further evaluating PFRP-collagen therapy for the treatment of recalcitrant ulcers.

## Figures and Tables

**Table I:** Patients characteristics, past medical history, ulcer site and wound category.

Patient	Age (years)	Gender	Patient condition at enrolment	Ulcer site	Volume or dimensions at enrolment	Wound category	Treatment period (weeks)	Healing situation
1	46	Male	Hypomobile	Sacrum	141 ml	UW	16	Healed
2	48	Male	Hypomobile	-	86 ml	TW	24	Healed
3	60	Male	Hypomobile	-	35 ml	UW	8	Healed
4	49	Male	Hypomobile	Sacrum	70 ml	UW	9	Healed
5	55	Male	Hypomobile	-	50 ml	UW	9	Healed
6	45	Female	Omphalitis	-	240 ml	UW	8	Healed
7	38	Male	Hypomobile	-	30 ml	TW	12	Healed
8	28	Male	Pilonidal sinus	-	204 ml	TW	8	Healed
9	30	Female	Pilonidal sinus	-	180 ml	TW	7	Healed
10	52	Female	Diabetic and hypomobile	-	5×4×1 cm	SW	12	>50% improvement

UW, undermined wounds; TW, tunneling wounds; SW, superficial wounds. Dimensions are provided as length × width × depth.
